# REMOTE‐AD: An Expert Group on Remote Digital Cognitive Assessment and Monitoring for Early Alzheimer's Disease

**DOI:** 10.1002/alz70856_102836

**Published:** 2025-12-24

**Authors:** David Berron, Stefano F Cappa, John Clarkson, Emrah Düzel, Jason J. Hassenstab, Frank Jessen, Roos J Jutten, Anja Kassner, Alexandra König, Sébastien Libert, Coco Newton, Kathryn V Papp, Sarah E. Polk, Silke Schicktanz, Michael Schöll, Sietske A.M Sikkes, Jochen René Thyrian, Wiesje M. van der Flier, Michael Wagner, Dennis Chan

**Affiliations:** ^1^ Clinical Memory Research Unit, Department of Clinical Sciences, Lund University, Lund, Sweden; ^2^ German Center for Neurodegenerative Diseases (DZNE), Magdeburg, Germany; ^3^ IUSS, Pavia, Italy; ^4^ University of Cambridge, Cambridge, Caambridge, United Kingdom; ^5^ Institute of Cognitive Neurology and Dementia Research (IKND), Otto‐von‐Guericke University, Magdeburg, Sachsen Anhalt, Germany; ^6^ Washington University in St. Louis, St. Louis, MO, USA; ^7^ University Hospital Cologne, Cologne, Germany; ^8^ Deutsches Zentrum für Neurodegenerative Erkrankungen e. V. (DZNE) Bonn, Bonn, Germany; ^9^ Alzheimer Center Amsterdam, Neurology, Vrije Universiteit Amsterdam, Amsterdam UMC location VUmc, Amsterdam, Netherlands; ^10^ RoX Health GmbH, Berlin, Berlin, Germany; ^11^ ki:elements GmbH, Saarbrücken, Germany; ^12^ Côte d'Azur University, Nice, France; ^13^ Alzheimer Europe, Senningerberg, Luxembourg, Luxembourg; ^14^ University College London, London, London, United Kingdom; ^15^ Brigham and Women's Hospital, Harvard Medical School, Boston, MA, USA; ^16^ University Medical Center Göttingen, Göttingen, Lower Saxony, Germany; ^17^ Department of Psychiatry and Neurochemistry, Institute of Physiology and Neuroscience, University of Gothenburg, Gothenburg, Västra Götalands län, Sweden; ^18^ Department of Neurodegenerative Disease, UCL Institute of Neurology, London, United Kingdom; ^19^ University of Gothenburg, Gothenburg, Västra Götalands län, Sweden; ^20^ Deutsches Zentrum für Neurodegenerative Erkrankungen e. V. (DZNE), site Rostock / Greifswald, Greifswald, Mecklenburg‐Vorpommern, Germany; ^21^ Institute for Community Medicine, University Medicine Greifswald, Greifswald, Mecklenburg‐Vorpommern, Germany; ^22^ Alzheimer Center Amsterdam, Neurology, Amsterdam UMC Location VUmc, Vrije Universiteit Amsterdam, Amsterdam, Netherlands; ^23^ German Center for Neurodegenerative Diseases (DZNE), Bonn, Germany; ^24^ Department for Old Age Psychiatry and Cognitive Disorders, University Hospital Bonn, Bonn, Germany

## Abstract

**Background:**

Remote digital cognitive assessments have great potential to address major unmet needs in global healthcare services. Early detection of Alzheimer's disease (AD) and the prediction and monitoring of disease progression represents an ideal use case given the scale of the disease and the limitations of legacy approaches. However, for remote digital assessments to be deployed at greatest benefit to both user and provider stakeholders, new frameworks for their use will need to be put in place to deliver best practice and maintain fitness for purpose into the future.

**Method:**

The expert group REMOTE‐AD, funded through the EU Joint Programme on Neurodegenerative Disease Research (JPND), brings together a multinational and multidisciplinary expert group to provide a framework and standards for the future use of remote digital cognitive assessments in early AD.

**Result:**

REMOTE‐AD is structured into four groups working on 1.) Determination of the unmet needs in AD clinical practice that can be addressed with remote digital assessments, 2.) Delivery of a framework for identification and appraisal of appropriate measures and measurement technologies for differing use cases, 3) Evaluation of social and ethical considerations relating to the deployment of remote digital assessments and 4) Design of systems for future implementation. Two workshops will be convened to outline scope of work and then to finalize recommendations. Here we will present results arising from the first workshop.

**Conclusion:**

Remote digital tools form part of the future of AD management but any plans for deployment are currently limited by the absence of frameworks and systems for their usage. REMOTE‐AD has been assembled to address this need and its outputs will benefit healthcare services in the EU and worldwide.

